# Productive Entry Pathways of Human Rhinoviruses

**DOI:** 10.1155/2012/826301

**Published:** 2012-11-26

**Authors:** Renate Fuchs, Dieter Blaas

**Affiliations:** ^1^Department of Pathophysiology, Medical University of Vienna, Währinger Gürtel 18-20, 1090 Vienna, Austria; ^2^Department of Medical Biochemistry, Max F. Perutz Laboratories, Vienna Biocenter, Medical University of Vienna, Dr. Bohr Gasse 9/3, 1030 Vienna, Austria

## Abstract

Currently, complete or partial genome sequences of more than 150 human rhinovirus (HRV) isolates are known. Twelve species A use members of the low-density lipoprotein receptor family for cell entry, whereas the remaining HRV-A and all HRV-B bind ICAM-1. HRV-Cs exploit an unknown receptor. At least all A and B type viruses depend on receptor-mediated endocytosis for infection. In HeLa cells, they are internalized mainly by a clathrin- and dynamin-dependent mechanism. Upon uptake into acidic compartments, the icosahedral HRV capsid expands by *~*4% and holes open at the 2-fold axes, close to the pseudo-3-fold axes and at the base of the star-shaped dome protruding at the vertices. RNA-protein interactions are broken and new ones are established, the small internal myristoylated capsid protein VP4 is expelled, and amphipathic N-terminal sequences of VP1 become exposed. The now hydrophobic subviral particle attaches to the inner surface of endosomes and transfers its genomic (+) ssRNA into the cytosol. The RNA leaves the virus starting with the poly(A) tail at its 3′-end and passes through a membrane pore contiguous with one of the holes in the capsid wall. Alternatively, the endosome is disrupted and the RNA freely diffuses into the cytoplasm.

## 1. Introduction

Human rhinoviruses (HRVs) are icosahedral (30 nm in diameter) and nonenveloped with a (+) ssRNA genome of ~7100 bases. Belonging to the family Picornaviridae, genus *Enterovirus*, they are composed of 60 copies each of four capsid proteins, VP1 to VP4. In 1987, HRVs from clinical samples were serotyped into 100 strains [1]. Recently, complete genome sequences of all known HRVs were determined. Phylogenetic analyses grouped them into 3 species; 74 HRV-A, 25 HRV-B, and 6 HRV-C [2]. Since then, many more rhinoviruses (mostly of type C) were identified in clinical specimens [3–5]. Independent from this classification, HRV-A and HRV-B are divided into two groups based upon the receptors exploited for host cell attachment; the minor receptor group, including the so far identified 12 HRV-A, bind low-density lipoprotein receptor (LDLR), very-LDLR (VLDLR), and LDLR-related protein 1 (LRP1) [6–9], while the remaining HRVA and HRV-B (constituting the majority, that is, the major group) use intercellular adhesion molecule 1 (ICAM-1) for cell entry [10]. Some major group HRVs (HRV8, 54, and 89) can also use heparan sulfate proteoglycans (HSPG) as an additional receptor [7, 11, 12] either as wild type (wt) or after adaptation to grow in cells lacking ICAM-1. This is achieved by numerous cycles alternating between blind passages and boosting in permissive cells [13, 14]. The receptor(s) for HRV-Cs is unknown [15].

Species A and B viruses are the cause of more than 50% of all mild infections of the upper respiratory tract known as the common cold [16]. The typical symptoms are inflammatory reactions of the nasal epithelium with the release of kinins, leukotrienes, histamine, interleukin 1 (IL-1), IL-6, IL-8, TNF-*α*, and RANTES [17]. HRV infections are usually benign and self-limiting, but recurrent, and therefore generate enormous economic costs. In 2001, the socioeconomic burden for noninfluenza virus-related respiratory infections due to expenses for medication and working days lost amounted to $40 billion in the USA alone [18, 19]. Since 1957 [20] evidence has been accumulating that HRVs are associated with asthma and wheeze by also infecting the lower airways. It is now well acknowledged that HRVs are involved in the exacerbations of asthma, cystic fibrosis, chronic obstructive pulmonary disease, pneumonia, sinusitis, otitis media, and wheezing of infants [21]. In addition to the costs detailed above, direct and indirect costs from such complications in asthmatics amount to $60 billion per year in the USA [22]. Global spending for respiratory infections can be estimated to be in the trillions of US dollars per year [18]. 

The recently discovered HRV-Cs appear to give rise to more severe respiratory tract illness especially in pediatric patients. HRV-C infections, in addition to symptoms of the common cold, cause pharyngitis, croup, otitis media, bronchiolitis, or pneumonia. This species must have circulated in the population for at least 10 years, but probably much longer, as they escaped detection because of being refractive to propagation in tissue culture [15]. The establishment of highly sensitive PCR methods now enables detection and strain typing within hours from clinical samples [3]. In hospitalized children HRV-Cs were also found in plasma, pericardial fluid, and stool samples [4, 23, 24] and the quite high concentrations question whether their replication is definitely limited to the respiratory system. Interestingly, the novel technologies detected HRV-As and HRV-Bs in fecal specimens as well. It is thus possible that all HRVs are not exclusively transmitted by the nasal/oral route but exploit a fecal-oral pathway as well. The viremia observed (preferentially) in HRV-C infections may be indicative for a distinct pathogenicity as compared to A and B viruses [16, 23]. 

At the time of writing, 148 HRV types had been found circulating in the human population [4]. Since recovery from infection with one serotype does not protect against reinfection with another serotype, vaccination appears difficult although common antigens have been identified [25–28]. A mixture of recombinant capsid proteins might thus be worthwhile to consider as a possible vaccine [28, 29]. Nevertheless, so far other means of disease prevention are believed to be more effective [30]. These include antiviral agents inhibiting either uncoating (by binding into a hydrophobic pocket within the capsid) or replication (by targeting virally encoded enzymes, such as the proteases). Such drugs are highly desirable for people suffering conditions like asthma where infection with a rhinovirus might heavily aggravate the symptoms. However, so far none of the initially promising compounds has reached clinical application. 

## 2. Overview of the HRV Life Cycle

 HRV infection typically involves the following sequence of events: (i) virus binding to the respective cognate receptors at the plasma membrane, (ii) entry into the cell by receptor-mediated endocytosis, (iii) transition from the native virus to a hydrophobic subviral particle, (iv) release of the viral RNA (uncoating), (v) RNA penetration into the cytoplasm, (vi) synthesis of viral proteins, (vii) RNA replication, and (viii) assembly and release of new, infectious virions. 

 Concomitant with HRV cell entry structural changes of the viral capsid occur that ultimately result in release of the genomic RNA. It is believed that native HRVs first lose the innermost capsid protein VP4 resulting in the generation of subviral A-particles [31]; these are further converted to (empty) B-particles after the release of the RNA. This view is supported by the finding that A-particles of the related poliovirus are infective, although at a substantially reduced rate [32]. Native virions and subviral particles can be separated by ultracentrifugation; native virions sediment at 150S, A-particles sediment at 135S, and empty B-particles have a sedimentation constant of 80S [33]. 

 Upon arrival of the viral genome in the cytosol the RNA is translated into a polyprotein that is autocatalytically and cotranslationally cleaved by the viral proteinases 2A^pro^, 3C^pro^, and its precursor 3CD^pro^, into structural proteins VP1, VP0, VP3, and the nonstructural proteins required for virus replication [34]. These include the RNA-dependent RNA polymerase 3D^pol^ as well as the precursor proteins (3CD, 2AB) that induce the formation of membrane vesicles derived from intracellular membrane-bounded compartments acting as scaffold for the RNA polymerase. Upon virus assembly maturation cleavage of VP0 into VP2 and VP4 occurs by an unknown protease [35].

## 3. HRV Receptors and Their Virus Binding Properties

In the following we will concentrate on the receptors for HRV-A and HRV-B since HRV-C receptors have not been identified. The physiological function and cell-type-specific expression of ICAM-1 and LDL-receptors are different. ICAM-1, a type1 transmembrane protein, is predominantly involved in cell-cell adhesion in endothelial cells and in immune reactions by binding to the integrins lymphocyte function antigen 1 (LFA1 i.e., CD11a/CD18) and macrophage 1 antigen (Mac1, i.e., CD11b/CD18) [36]. Its extracellular part is composed of five typical immunoglobulin-like domains [37] and its short cytoplasmic tail has no known clathrin-coated pit localization signals. In cryo-EM 3D-reconstructions of complexes between soluble recombinant fragments of ICAM-1 and HRV3, HRV14, and HRV16, its first domain is seen to contact the virus inside the canyon, a cleft encircling the dome at the vertex, whereas the other domains protrude from the surface [38–41]. 

As will be detailed below, the structural alterations of the viral capsid of major group viruses during uncoating are catalyzed by ICAM-1 in a pH- [42] and temperature-dependent manner [43, 44]. This is similar to poliovirus where receptor binding leads to uncoating [45]; however, the structural changes of poliovirus are independent of low pH [46–49]. In contrast to ICAM-1 and the poliovirus receptor, members of the LDLR family only function in ligand internalization but do not catalyze uncoating. They transduce signals and undertake multiple rounds of recycling to the plasma membrane and reinternalization [50]. Their ligand-binding domains are composed of different numbers of ligand-binding (type A) repeats (LDLR has 7, VLDLR has 8, and LRP1 has 31). In LDLR, they are at the N-terminus and followed by three regions with similarity to the epidermal growth factor precursor (EGF-domain) containing YWTD motives forming a 6-bladed *β*-propeller, a domain with O-glycosylation proximal to the membrane, a transmembrane domain, and a cytoplasmic tail with an NPXY internalization motive [51, 52]. The other members of the LDLR family exhibit similar domain arrangements [53, 54]. In tissue culture cells LDLR and LRP1 mediate productive entry of minor group viruses [6]. It is likely that this also holds true for VLDLR as its downregulation inhibits infection with minor group HRVs [55]. As demonstrated for HRV2 and a recombinant fragment of VLDLR, the ligand binding repeats attachment to the top of the star-like mesa at the vertex at the five-fold axis of symmetry in a multimodular manner; thus, the binding site is distinct from that of ICAM-1 [56–59]. As mentioned above, minor group virus uncoating is receptor independent and might even be inhibited to some extent by the bound receptor; presumably, it prevents movements of the five copies of VP1 that occurs during conversion to the subviral particle [60, 61]. 

**Figure 1:**
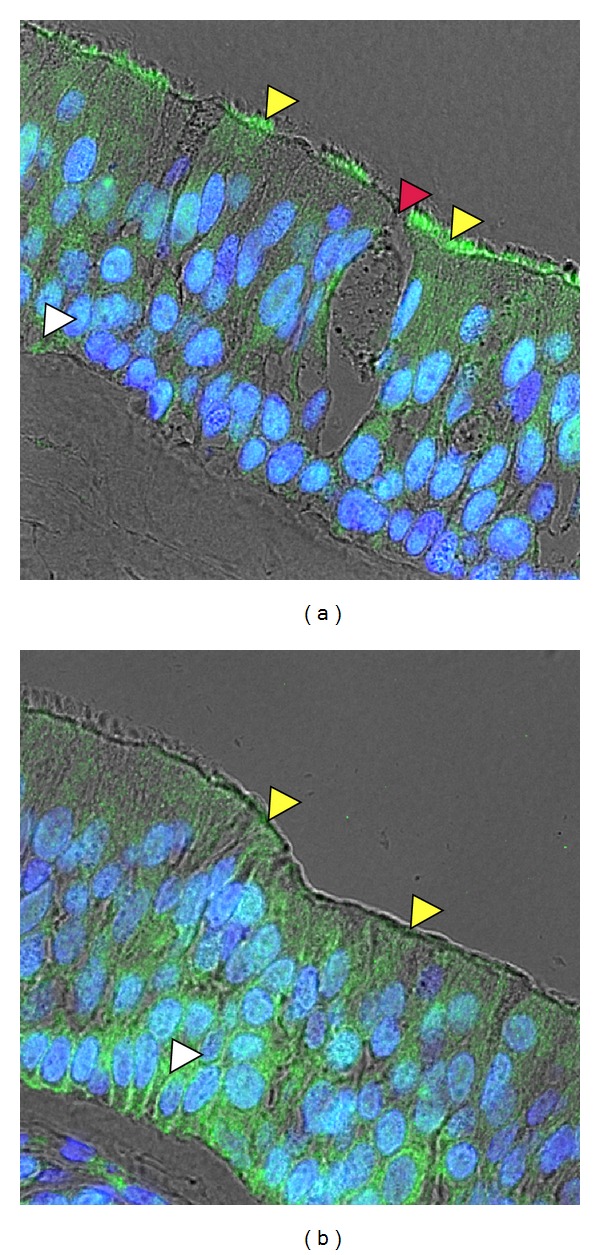
Receptors for minor group viruses are expressed in nasal epithelial cells. Paraffin-embedded nasal tissue sections were prepared, rehydrated, and subsequently incubated with anti-LDLR [60] or anti-LRP1 [70] antibodies, followed by the respective fluorophore-labeled antichicken or antirabbit Alexa-488-IgG. Nuclei were stained with Hoechst dye. Pictures shown are overlays of immunofluorescence and phase contrast images. LDLR (a) and LRP1 (b) are present at the apical (yellow arrow heads) surface in ciliated as well as in basal cells (white arrow heads). A mucous secreting cell is indicated by a red arrow head.

HRVs replicate in (polarized) epithelial cells in the upper and lower airways [62–64]. As known for HRV-A and -B viruses, only a few ciliated cells become infected and this without obvious cytopathic effect. This is in agreement with the receptor for major group viruses, ICAM-1, being expressed in only 1% of these cells in nasal tissue in the absence of inflammation [65, 66]. The very low level of ICAM-1 expression in highly differentiated ciliated epithelial cells, as compared to undifferentiated basal cells, appears to limit infection [67, 68]. No such *in vivo *data are available for minor group HRVs. Immunostaining for LDLR and LRP1 of normal nasal tissue revealed the presence of the receptors at the apical surface in ciliated as well as in basal cells. As shown in Figure 1, receptor expression between individual ciliated cells varies considerably. Neither the expression of ICAM-1 nor of LDLR/LRP1 in the nasal epithelium has been quantified *in situ.* The polarity of ICAM-1 expression is also unknown. Receptor expression obviously determines the amount of virus that can bind and enter the cells. However, the presence of a suitable receptor is not sufficient for productive infection, as subsequent events such as entry, uncoating, RNA penetration into the cytoplasm, and replication must also function properly in a given cell [69].

## 4. HRV Structure and Capsid Alterations during Uncoating

 As all picornaviruses, HRVs possess *T* = 1,  *P* = 3 icosahedral symmetry with 60 copies of each of the capsid proteins VP1, 2, 3, and 4. Except from the small internal myristoylated VP4, they fold similarly into a beta-barrel whose eight antiparallel beta-sheets are connected with long (external) and short (internal) loops. The former are exposed and make up the antigenic sites, targets for type-specific antibodies [71–76]. The inner capsid wall is stabilized by an intricate network built by interacting residues of the N-terminal extensions of VP1 and VP2 under contribution of the backbone of VP3; residues of both VP1 and VP2 interact with the RNA [77].

Concomitant with HRV cell entry the viral capsid suffers structural alterations. Induced by interacting with ICAM-1 (major group HRVs, [44, 78, 79]) or exclusively triggered by the low pH (minor group HRVs) the virus loses the pocket factor, presumably a fatty acid residing in a void within VP1, and expands by about 4% [80, 81]. In major group viruses, depending on the serotype, the low pH may aid the “catalytic” function of ICAM-1 [42]. For HRV2, it was recently shown that on expulsion of the pocket factor, the empty space allows for Met^213^ of VP1 to move in. This results in a substantial part of the chain pivoting over this site [80]. The final result is a loosing of intra- and intersubunit interactions and the opening of three types of holes [77]; the largest ones at the twofold axes and smaller ones at the pseudo three-fold axes and at the base of the star-shaped domes at the vertices. The small innermost myristoylated capsid protein VP4 escapes (its exit site is unknown but the holes are big enough for an unfolded protein to pass) and N-terminal sequences of VP1 become exposed most probably on exiting through the pore close to the pseudo-threefold axes [77]. A similar exit point has been proposed earlier for HRV3 [38] and poliovirus [82–84]. Due to the amphipathic nature of the N-terminal VP1 sequences these A-particles then attach to endosomal membranes and release the RNA. Native virus is thereby converted into B-particles (i.e., empty capsids). 

Recent cryo-EM data demonstrated that the RNA is much more structured than previously thought; in addition to the well-known contacts with the conserved Trp^38^ of VP2 ([85] and references therein) it also interacts with residues of VP1. These latter are part of an interaction network contributed by the N-terminal extensions of VP1 and VP2. Comparison of the 3D X-ray structures of native HRV2 and its empty capsid and a model of the 135S-particle at close to atomic resolution reveals that this network is broken in the subviral particles [77]. Since a number of acid-sensitive residues are nearby it is likely that their protonation weakens this network letting go the RNA to escape through one of the holes. In A-particles the RNA-protein contacts have changed with respect to the native virion; those at the 2-fold axes are maintained but new ones are being established with N-terminal residues of the VP3 ß-cylinder that have become accessible after VP4 has escaped. 

## 5. HRV Entry Pathways and Intracellular Trafficking

### 5.1. Entry into Tissue Culture Cells

Early on, the low pH sensitivity of HRVs suggested cell entry by endocytosis and uncoating in endosomal compartments [31, 33, 86]. Endocytosis, the uptake of extracellular material within membrane-bound vesicles, has first been described by Metschnikoff about 130 years ago [87]. It starts by binding of ligands to specific receptors, concentration of these complexes in specialized domains at the plasma membrane (e.g., coated pits, caveolae, and lipid rafts) followed by membrane invagination and pinching off of primary endocytic vesicles. Viruses have proven to be valuable tools for studying the mechanisms of primary endocytic vesicle formation [88]. So far, clathrin-mediated endocytosis is best characterized [89]. It depends on particular sequence motives in the cytoplasmic tail of the transmembrane receptors for clathrin-coated pit formation [90]. Caveolae-dependent as well as clathrin- and caveolin-independent pathways are less defined. In addition to specific coat proteins (clathrin, caveolin, flotillin) and accessory molecules (adaptor proteins), the GTPase dynamin plays an essential role in the constriction/fission process during clathrin-, caveolin-, and lipid-raft-mediated uptake [91]. Based on the distinct requirements for clathrin, caveolin, dynamin, cholesterol, and various other accessory molecules, 10 different endocytic pathways have been differentiated so far [88]. Irrespective of the uptake mechanism, internalized receptors and ligands are first delivered to early (sorting) endosomes [92–95]. From early endosomes, internalized material can then follow different intracellular routes [95, 96]: (i) transport to lysosomes, resulting in degradation of ligands and certain receptors, (ii) recycling to the cell surface, and (iii) in polarized cells, transport from one plasma membrane domain to the opposite plasma membrane domain (transcytosis).

**Figure 2:**
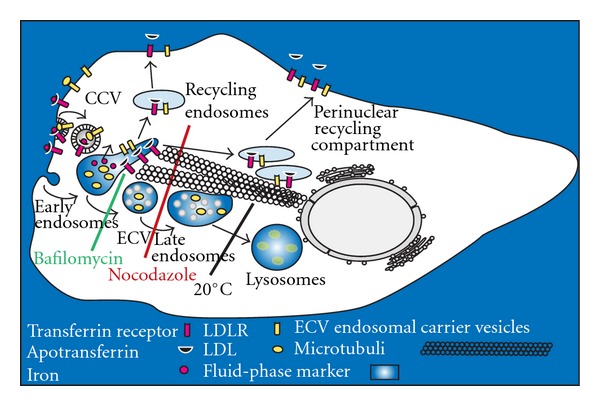
Influence of bafilomycin, nocodazole, and low temperature on endocytic pathways in HeLa cells. The recycling pathway (transferrin, LDLR) and the transport of ligands (LDL) and fluid-phase marker to lysosomes are shown. Iron-loaded transferrin binds to its receptor at the plasma membrane. The complex is internalized via clathrin-coated vesicles (CCV) and delivered into early endosomes within 2–5 minutes, where the iron is released and transferred into the cytoplasm. Apotransferrin remains bound to the receptor and recycles via a fast and a slow pathway. At the plasma membrane, apotransferrin is released at the neutral pH. Similarly, internalized LDL is released from its receptor in early compartments allowing for LDLR to return to the plasma membrane via the same pathways as apotransferrin. Although a major portion of fluid-phase marker (e.g., dextran) is recycled, the remaining fluid and released ligands (LDL) are delivered from early endosomes (within 5 min), via endosomal carrier vesicles (ECV) and late endosomes (within 15 min), to lysosomes (within 25 min). Transferrin transport to and recycling via the perinuclear recycling compartment is blocked by nocodazole, whereas bafilomycin and lowering the temperature to 20°C are without effect. In contrast, bafilomycin arrests fluid-phase markers in early endosomes by preventing budding of ECV, whereas nocodazole leads to accumulation of cargo in ECV. Finally, incubation at 20°C prevents delivery of markers from late endosomes to lysosomes. For further details and endosomal pH determination see [105, 108]. Adapted from Fuchs and Blaas [112].

A main feature of endosomes is their ability to acidify their interior by a vacuolar proton ATPase (V-ATPase) [97–99]. Due to the concerted action of the V-ATPase, Na^+^/K^+^-ATPase, transporters, and ion channels, distinct pH values are established in endocytic subcompartments that play an important role in trafficking of macromolecules through endocytic pathways, in ligand degradation, and inactivation of internalized pathogens [100, 101]. Although endocytic-coated vesicles may not be acidic [102], the mildly acidic pH in early endosomes causes the dissociation of many ligands from their receptors [103] allowing for receptor recycling; a small fraction of internalized fluid containing the released ligands is routed through late endosomes to lysosomes for rapid degradation (Figure 2). The formation of “nascent” late endosomes may involve budding and fission events from early endosomes resulting in “endosomal carrier vesicle” (ECV) formation [104]. In any case, nascent late endosomes undergo a sequence of maturation events until fusion with lysosomes can take place [101]. Material *en route* to lysosomes (pH 4.5–4.0) is exposed to an increasingly acidic pH during transport from early endosomes (pH 6.5–6.0) through ECV/late endosomes (pH ≤ 5.6) [100, 105–108]. Concomitantly with the decrease in pH the internal ionic milieu of endosomes undergoes major alterations as compared to the outside environment [99]; the calcium and chloride concentration first decreases and subsequently increases, and the continuous decrease in sodium ions is paralleled by potassium ion increase. These alterations are brought about by ATPases, transporters, channels, and passive ion permeabilities in the membrane of endosomes. 

Receptors (e.g., the transferrin receptor, LDLR), certain ligands (e.g., transferrin), plasma membrane proteins, and the majority of internalized fluid are recycled to the cell surface [95]. As exemplified by transferrin, recycling can occur via two pathways; from early endosomes with *t*_1/2_ ≈ 2 min (fast) and from the perinuclear recycling compartment (PNRC) with *t*_1/2_ ≈ 12 min (slow) [109, 110]. In CHO and Hep2 cells, the pH of the PNRC is higher than that of early endosomes, whereas it is more acidic than in early endosomes in HeLa cells [95, 105]. 

Due to distinct mechanisms of endosomal transport to lysosomes and recycling to the plasma membrane, different drugs and dominant negative mutants, for example, of rab GTPases, may be used to arrest ligands, receptors, and fluid in specific endosomal subcompartments (see Figure 2). Furthermore, when ligands destined to lysosomes are taken up at reduced temperature (e.g., at 20°C), internalization takes place (albeit at reduced rate) and sorting does occur but fusion of late endosomes and lysosomes is prevented [111]. In contrast, the kinetics of transferrin endocytosis and recycling is unaffected at 20°C [105]. Consequently, these treatments are valuable tools for investigating whether viruses follow a recycling or degradative pathway and to identify the compartment where virus penetration/uncoating takes place.

One particular virus can use multiple entry pathways [113–115], but not all may result in delivery to the compartment where the internal milieu allows for the structural alterations and genome release (i.e., for productive uncoating) leading to infection of the host cell. Why is it important to define the virus entry route and the compartment of productive uncoating and genome penetration? Since distinct cellular factors are necessary for virus internalization and intracellular routing these molecules may represent potential drug targets for antivirals. For example, Urs Greber's group recently demonstrated that niclosamide, an antihelminthic drug approved by the FDA since a long time, prevents infection by various HRVs via neutralizing acidic endosomes [116]. Another example is HIV; this enveloped virus penetrates into the cytoplasm by fusion with the plasma membrane but it can be routed into an unproductive pathway by enhancing its endocytosis [117, 118]. Redirection from a productive to an unproductive endocytic compartment was also shown for coxsackievirus B3, another picornavirus [119]. 

Combining small molecule inhibitors, dominant-negative mutants, RNAi, immunofluorescence microscopy, FISH, and subcellular fractionation with infectivity assays, we have been studying the productive entry route(s) of HRVs into tissue culture cells [12, 108, 120–125]. Because of the high particle to infectious particle ratio (between 24 : 1 and 240 : 1; [126] or even much higher [127]) determining infectious virus was and is of particular importance in such investigations. 

**Figure 3:**
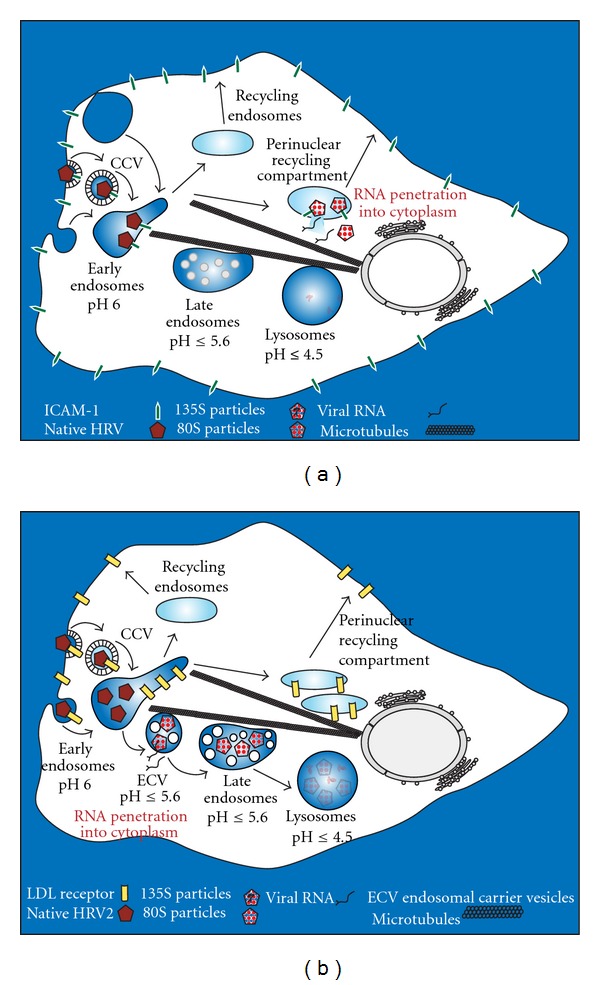
Entry, intracellular trafficking, and uncoating of HRVs in HeLa cells. (a) The major group virus HRV14 is internalized via clathrin-mediated endocytosis and delivered into early endosomes from where it presumably further traffics into the perinuclear recycling compartment. Structural modification of the viral capsid catalyzed by ICAM-1 is probably coupled to RNA release and rupture of the endosomal membrane. These events lead to delivery of free RNA and empty capsids into the cytoplasm. Most likely, uncoated virus and the RNA escape from the perinuclear recycling compartment. (b) HRV2 enters via clathrin-dependent and independent pathways and dissociates from its receptors at mildly acidic pH in early endosomes. Receptors are recycled and HRV2 is transferred to ECV/late endosomes where the more acidic pH (≤5.6) induces the structural modification resulting in uncoating and RNA transfer into the cytosol. Finally, residual native virus, subviral particles, and viral RNA are transported via late endosomes to lysosomes where they are degraded. Adapted from Fuchs and Blaas [112]. Note that the pathways might differ in other cell types.

The entry mechanism of major group HRVs appears to by cell-type specific. In HeLa cells (Figure 3(a)), HRV14 uptake occurs by a dynamin- and presumably clathrin-dependent route [128, 129], whereas endocytosis in ICAM-1 overexpressing rhabdomyosarcoma cells is independent of clathrin, caveolin, flotillin, and lipid rafts [123]. HRV14 endocytosis and productive uncoating in these cells were partially inhibited by blocking dynamin function with dynasore and by disrupting the actin cytoskeleton. Conversely, the Na^+^/H^+^ exchange inhibitor amiloride prevented uptake and uncoating indicative for macropinocytosis as infectious entry route [123]. Some major group viruses (HRV8, HRV54, and HRV89) either use or can be adapted to use HSPG as an alternative receptor for cell binding in addition to ICAM-1 [11–13]. Entry and infection of the HSPG-binding variant of HRV8 (HRV8v) in rhabdomyosarcoma cells devoid of ICAM-1 were very similar to entry and infection of HRV14 in these cells [12]. Our recent studies on the productive entry route of HRV89, another major group virus, in HeLa cells are in agreement with a clathrin- and dynamin-dependent mechanism [130]. This is surprising since ICAM-1 has no known clathrin-coated pit localization signals in its cytoplasmic tail and GPI-linked ICAM-1 can mediate HRV14 internalization and infection [131]. After plasma membrane binding, HRV14-ICAM-1 complexes are delivered into mildly acidic early endosomes [124, 132]. It has not been investigated *in vivo* whether major group HRVs dissociate from ICAM-1 at low endosomal pH, as suggested from *in vitro* experiments [133]. Neither is the further trafficking of major group HRVs in HeLa cells completely clear. By using immunofluorescence microscopy, HRV14 was found in early and late endosomes when internalized at 20°C [124] but not when uptake occurred at 34°C. Under the former condition the conformational modification of the capsid catalyzed by ICAM-1 and thus infection is inhibited. These results contradict recent data by Khan et al. [123] where HRV14 exhibited some colocalization with the fluid-phase marker dextran after co-internalization at 34°C in ICAM-1 overexpressing rhabdomyosarcoma cells. Whether this discrepancy is due to ICAM-1 overexpression or the different cell type remains to be demonstrated. As already shown by Lonberg-Holm and Korant [31] the virus is apparently not transported to lysosomes as viral RNA is not degraded after 60 min incubation at 34°C. Collectively, these data may indicate that HRV14 follows the recycling pathway, is targeted to other organelles, or disrupts the endosomes for escape (Figure 3(a)).

The so far investigated major group HRVs were found to be dependent on ICAM-1 for capsid modification; some HRVs are additionally “primed” by low endosomal pH for receptor-catalyzed uncoating [42]. Infection by these serotypes is either completely (HRV16), partially (HRV14), or not (HRV3) blocked by raising the endosomal pH with bafilomycin [42, 132]. Inability of bafilomycin to completely block infection by major group HRVs would indicate RNA uncoating/penetration in/from early endosomes or the PNRC since this drug not only increases endosomal pH but also halts transport of cargo to late endosomes [134]. In contrast, transfer from early endosomes to the PNRC persists in the presence of bafilomycin [105, 135].

HRV2, a prototype minor group HRV, is internalized by clathrin-mediated endocytosis into early endosomes (Figure 3(b)) [125]. However, when this pathway is blocked by cytosol acidification or overexpression of nonfunctional dynamin, HRV2 enters via a clathrin- and dynamin-independent pathways [122, 136]. Similar to the natural ligand LDL the virus dissociates from its receptors in early endosomes. Obviously, the specific internal milieu in these compartments (low calcium) facilitates dissociation by destabilizing the conformation of the ligand-binding repeats. This is supported by the finding that little LDL and HRV2 are released from the receptors at the plasma membrane at pH 6.5–6.0 [52, 60, 137]. Furthermore, LDLR and HRV2 release depend on intramolecular competition with the beta-propeller domain of the receptor [60]. LDLR is recycled to the plasma membrane [60] and the virus is targeted via typical LAMP-positive late endosomes to lysosomes where it is rapidly degraded [31, 138]. Impeding HRV2-LDLR dissociation by deletion of the beta-propeller and the EGF-C domain of human LDLR results in degradation of the entire complex and thus in receptor downregulation [60]. In contrast to major group HRVs and their receptor ICAM-1, uncoating and membrane penetration can take place in the absence of LDLR/LRP and this process is solely dependent on pH ≤ 5.6 *in vitro* and *in vivo *[139, 140]. In accordance with this pH requirement, HRV2 has been found to release its RNA in ECV/late endosomes *in vivo *[124, 138]. 

### 5.2. Entry into Airway Epithelial Cells

Although HRV replication in HeLa cells and in primary human bronchial epithelial cells is comparable [141], HeLa cells are not a valid model for the airway epithelium. The respiratory epithelium is built from different cell types; predominantly ciliated columnar (epithelial) cells and mucous-secreting goblet cells (Figure 1). Both are polarized with their apical and basolateral plasma membrane separated by tight junctions. The basal cells are small and rounded and are in contact with the basal lamina; they can differentiate into the other cell types [142]. For studying the mechanisms of virus replication outside the human body either organ cultures of biopsy material [143] or cultures of primary nasal, tracheal cells, bronchial epithelium [144], or immortalized airway cell lines (Calu3, 16HBE) [145] were used. Since no comparative studies on the productive entry pathways of HRVs in all these systems have been carried out, it is unknown which cell line or cell type would best represent the *in vivo* situation.

The clinical symptoms of an HRV infection are the consequence of the immune response of the infected respiratory epithelium. Thus, it is apparent that HRV binding to its receptor and virus entry activate various signaling pathways leading to secretion of inflammatory mediators. The nonreceptor protein tyrosine kinase Syk has been identified as an early signaling molecule that ultimately leads to IL-8 expression [146, 147]. Upon binding of HRV16 to ICAM-1 in primary bronchial epithelial cells Syk is recruited to the plasma membrane together with ezrin that in turn can interact with filamentous actin. Both Syk and ezrin associate with clathrin in response to virus binding. These data suggest—in agreement with studies in HeLa cells—that the major group virus HRV16 enters via a pathway involving clathrin and actin. Following virus entry, Syk and ezrin appear to redistribute from the plasma membrane to endosomal compartments [148] suggesting that they might continue signaling from endosomes. Syk recruitment to ezrin also results in activation of the p85 regulatory subunit of the phosphatidylinositol (PI) 3-kinase and the Akt signaling pathway [147, 149]. Another target activated by binding of the major group virus HRV39 to ICAM-1 that is also involved in regulation of IL-8 expression has been shown to be Src [149, 150]. Src functions as an upstream regulator of p110*β* catalytic subunit of PI 3-kinase (that in turn forms a complex with p85 PI 3-kinase) and of Akt. Furthermore, HRV39 internalization and/or intracellular trafficking appear to depend on PI 3-kinase activation [149]. 

In contradiction to the observations described by Lau et al. [148], Dreschers and coworkers noticed the induction of ceramide-enriched membrane domains by major group and minor group viruses in nasal mucosa, isolated nasal epithelial cells, HeLa cells, and fibroblasts [151, 152]. Such domains may be important for p38-MAPkinase activation in response to HRV14 infection [153] as well as for HRV39-induced Src signaling in airway cells [150]. Remarkably, viral replication is not required for activation of p38-MAPkinase. Thus, most likely, receptor clustering by the multivalent virus on the one hand induces signals facilitating virus entry and/or replication and, on the other hand, signals upregulating the immune response. 

Despite considerable information on induction of inflammatory mediators by minor group viruses and inhibition of infection by various drugs, essentially no data have been published on the mechanism of entry and uncoating of minor group HRVs in airway cells. *In situ*, infection by major group HRVs leads to inflammation and increased ICAM-1 expression [66]. It is thus interesting that LDLR expression was found to be upregulated upon infection with the major group virus HRV14 or the minor group virus HRV2 in primary cultures of human tracheal epithelial cells [144]. *Vice versa*, in the same cells, ICAM-1 expression was also increased after infection with HRV14 or HRV2 [154]. These effects may be explained by HRV-induced activation of nuclear factors SP1 and (NF)-*κ*B that regulate both ICAM-1 and LDLR expressions. 

## 6. Mechanism of RNA Uncoating 

### 6.1. Penetration and Uncoating of Major Group Viruses

Although major group HRVs bind ICAM-1 at 4°C, the receptor-catalyzed structural modifications required for RNA release only occur at temperatures ≥26°C *in vitro* (shown for HRV3) [44] as well as *in vivo* (HRV14)[155]. In agreement with the inability of ICAM-1 to induce virus uncoating below 26°C, HRV14 was found in endosomes when internalized at 20°C. Although the pH in this compartment is about 5.6 [138] this is not sufficient for HRV14 uncoating in the absence of the receptor [156]. However, when the virus was internalized at 34°C, a condition where the viral RNA is released, HRV14 was not detected in endosomal compartments. We thus concluded that it penetrates into the cytoplasm by rupturing the endosomal membrane [124]. Further evidence for this mechanism was derived from different experimental approaches; (i) by electron microscopy, free HRV14 was seen in the cytosol 30 min after entry [129]. (ii) HeLa cell endosomes were labeled with the pH-sensitive FITC and the pH-insensitive Cy5-dextran. FACS and single-organelle flow analysis (SOFA) [120] demonstrated an increase in the mean pH from 6.0 (control, in the absence of virus) to 7.0 upon co-internalization of these fluid-phase markers with HRV14 (see Figure 3 in [112]). This correlated with a reduction in the number of the labeled endosomes by 23% indicating that the fluorescent markers had been released into the pH neutral cytoplasm. For comparison, adenovirus was analyzed in parallel. This virus is known to penetrate into the cytosol by very efficient endosome lysis, reducing the number of fluid-phase marker-labeled endosomes by 37%. At least in HeLa cells, uncoating and subsequent infection of HRV14 can also take place when the endosomal pH is neutralized by bafilomycin [132]. This drug, in addition to inhibition of V-ATPases, arrests markers en route to lysosomes in early endosomes [134]. In its presence HRV14 was not detected in isolated endosomes suggesting that the virus penetrates into the cytosol by rupture of early endosomes. Since the recycling pathway is not affected by elevating the endosomal pH it might be also considered that the virus penetrates from and ruptures the PNRC [105, 135]. Nevertheless, as long as the RNA has not been traced on its way from within the intact capsid into the cytosol, the localization of the process will remain indirect and lacking definite proof.

### 6.2. Uncoating and RNA Penetration of HRV2

In contrast to HRV14, HRV2 was localized in ECV/late endosomes in intact HeLa cells by immunofluorescence microscopy as well as by subcellular fractionation of isolated endosomes [124, 157]. Various experimental setups support a mechanism where the RNA is transferred from these compartments into the cytoplasm through a pore in the membrane [120, 121, 124]. As recently shown, under conditions of productive uncoating, HRV2 induces ion permeable channels presumably lined by viral proteins (see Figure 6 in [112]). Most likely, the RNA travels through these pores into the cytosol. RNA transfer is stimulated by a *trans*-negative membrane potential (endosome interior positive) as compared to inside positive potential but is unaffected by the pH gradient between endosomes and cytoplasm [158]. 

 Taken together, at present, data on rhinoviruses and poliovirus from different laboratories support a model in that VP4 and the amphipathic N-terminal extensions of VP1 insert into the membrane of late endosomes to form an ion-conducting pore. This channel would be exploited by the RNA to pass. The previous, quite suggestive model positioned the exit site of the RNA at a fivefold axis; this places five receptor molecules and five copies of the VP1 N-terminal extensions upright onto the membrane. However, according to the present model RNA exit occurs at a twofold axis leaving us with the question of how this opening in the viral shell can be positioned on the membrane (by receptors and/or the amphipathic extensions of the capsid proteins of the subviral particle) in a way as to form a contiguous channel. 

 RNA exit from poliovirus inside the cell has been localized to endosomal compartments by life cell microscopy [159] and membrane penetration of the viral genome has been demonstrated in intact liposomes on acidification of bound HRV2 [140]. Nevertheless, direct visualization of the RNA passing through this putative channel is lacking. 

In contrast to a number of older textbook illustrations, at least *in vitro*, the RNA leaves the virion with its 3′-end first and not with the 5′-end carrying the peptide VPg [160]. Since the 5′-end is being synthesized first, encapsidation is likely to start with this end and might terminate with the poly-(A) tail left close to the location where a hole is going to open at one of the 2-fold axes when the subviral particle forms [161, 162]. 

## 7. Future Perspectives

Although considerable progress has been made in unraveling the entry mechanisms of rhinoviruses into tissue culture cells, amazingly little is known on the entry route leading to productive infection of the airway epithelium. Identification of receptors for HRV-C type viruses, characterization of their entry pathways, and comparison with A and B types will shed light on the distinct pathology of infection caused by this virus species. A systems biology approach in combination with new high-throughput technologies may lead to identification of cellular host factors essential for HRV entry, trafficking, uncoating, signaling, and replication and thus point to novel drug targets [163, 164]. Structural studies using electron microscopy and X-ray crystallography of viruses and virus-(receptor)-liposome complexes in combination with novel technologies have shed light on the capsid modifications occurring during uncoating and the mode of RNA exit. Such techniques need now to be utilized to study the *in vivo *situation, that is, to characterize the molecular mechanisms of virus-endosome interaction and to visualize the RNA during transit from the protective viral shell through a membrane into the cytosol.
